# Crystallography and large research infrastructures, a perfect marriage

**DOI:** 10.1107/S2052252514019460

**Published:** 2014-08-29

**Authors:** Sine Larsen

**Affiliations:** a Department of Chemistry, University of Copenhagen, Universitetsparken 5, Copenhagen, DK-2100, Denmark

**Keywords:** large research infrastructures, editorial, neutron facilities, synchrotron facilities

## Abstract

The role of large-scale neutron and synchrotron facilities in the development of crystallographic research is discussed.

In this International Year of Crystallography (IYCr2014), it is natural to look back and reflect on the historical development of crystallography. Since the seminal experiments of Max von Laue and the Braggs (father and son) in 1912, X-ray tubes have been the dominant source for generating the short wavelength electromagnetic radiation (X-rays) used for crystallographic experiments. But in 1947, 35 years after the birth of modern crystallography, two important experiments were reported that in the longer term would have a great impact on crystallography and offer new opportunities for the development of the science. 1947 was the year when the first pioneering neutron diffraction experiments on NaH and NaD were reported by W. L. Davidson, G. A. Morton, C. F. Shull and E. O. Wollan (Davidson *et al.*, 1947 ▶). They showed that the elastic scattering of neutrons, discovered by J. Chadwick in 1932, could be used just like X-rays to provide complementary structural information on solids. The significance of this result was recognized in 1994 when C. F. Shull was awarded the Nobel Prize in Physics with B. N. Brockhouse. Another equally significant publication authored in 1947 by F. R. Elder, A. M. Gurewitsch, R. V. Langmuir and H. C. Pollock (Elder *et al.*, 1947 ▶) was on the light, the electromagnetic radiation, that emerged from the operation of the General Electric synchrotron accelerator.

It was immediately realised that the atomic scattering of neutrons offered new possibilities relative to those offered by X-rays for the study of materials, *e.g.* for the identification and location of hydrogen atoms in all types of materials, also in the presence of heavier atoms. The magnetic moment of the neutron paired with its lack of charge make neutrons the ideal experimental tool to study magnetism. Neutrons for materials research were first provided by nuclear reactors, and benefitted from the development of nuclear energy. Of the new neutron sources that have been constructed or are under construction, the majority are accelerator-based and generate neutrons by the spallation process. For both types of neutron sources, safety is an important issue. Although neutron diffraction experiments are in principle very similar to X-ray diffraction experiments, in practice they have been more complex to carry out due to limited access to large neutron facilities, and the requirement for large samples. However, neutron sources offer unique technical facilities that can be used to modify the sample environment.

The experimental use of synchrotron radiation, which initially was considered a nuisance by the particle physicists, developed at a much slower pace. The parasitic use of the radiation from particle accelerators contributed to the development of synchrotron-radiation-based science and promoted the demand to have dedicated so-called third-generation facilities. It should be noted that it took more than 40 years before funding could be obtained to construct the first third-generation synchrotrons. At this time the synchrotron facilities could benefit from the technological development at the neutron sources, since the experiments at synchrotrons are complementary and similar to those performed with at neutron sources.

At present it is possible for researchers to perform experiments on more than 20 neutron sources and close to 50 synchrotron facilities worldwide, and one may ask the question, how has the availability of these large research infrastructures influenced crystallographic research. The high brilliance of synchrotron radiation has naturally enabled the study of smaller and weakly diffracting samples like protein crystals. Protein crystallography is a field that has benefitted tremendously from access to synchrotron facilities, and it is fully justified to state that synchrotron radiation has revolutionized structural biology. The exponential increase in depositions of protein structures to the Protein Data Bank would not have been possible without the use of the highly automated beamlines for macromolecular crystallography at the synchrotrons. The number of protein structures deposited the since 2003 has more than doubled from 3921 to 8539 in 2013, and of the latter more than 90% were based on synchrotron data. The wider use of synchrotron radiation has had impact on the complementary use of neutron sources, and led to closer interactions between the X-ray and neutron scattering communities. The large facilities also play a role in the development of instrumentation that enables unique experiments with demanding sample environments. It has been interesting to see how this development has influenced commercial X-ray equipment, such that it is now possible to measure diffraction data in a laboratory that are of comparable quality to synchrotron data measured a few years ago.

It is important to note that access to the large-scale neutron and X-ray sources has led to a significant enlargement of crystallographic research. As well as traditional diffraction experiments for structure determination, neutrons and synchrotron radiation are now used to measure diffuse scattering, small-angle scattering and measurements by different spectroscopic techniques. These techniques are employed, independently or in combination, in many new research areas, as demonstrated by the growing number of IUCr Commissions (http://www.iucr.org/iucr/commissions) that now cover such new aspects of crystallography. The scientific development of crystallography is represented by the IUCr Commissions, and it is noteworthy that the activities of the majority of the Commissions now depend on the use of the large neutron and X-ray facilities. It has primarily been the use of neutron sources that has played a role in the study of charge, spin and and momentum densites. However, as demonstrated in the recent feature article in **IUCrJ** (Jørgensen *et al.*, 2014 ▶), it is now possible, using powder diffraction data measured with synchrotron radiation, to obtain information on the charge density of diamond.

Other **IUCrJ** papers are also good examples of how the use of neutrons and synchrotron radiation has penetrated crystallographic research. Synchrotron radiation has formed the basis of many of the experiments reported in the papers in **IUCrJ**, and is routine for protein structure determination. Radiation damage is destructive for the success of synchrotron radiation in protein crystallography, and significant efforts have been invested in software developments to deal with radiation damage. A potential breakthrough on how to handle this problem was seen in the publication by Stellato *et al.* (2014 ▶), in which they describe how they were able to measure room temperature radiation-damage-free diffraction data with synchrotron radiation using serial crystallographic measurements on thousands of lysozyme microcrystals. This breakthrough, which was initiated using methods developed for experiments with free electron lasers, is an excellent example of the fruitful cross-fertilization seen between experiments conducted at the different large research infrastructures. It is my hope that this cross-fertilization will continue in the future, contribute to the development of crystallography and lead to excellent crystallographic results published in **IUCrJ**.
